# Optimizing Complete Denture Fabrication With a Hybrid Analog–Digital Workflow: A Case Report

**DOI:** 10.1155/crid/6761654

**Published:** 2025-10-03

**Authors:** Edgar García Zea, Andres Ullauri, Pablo Lenin Benitez Sellan

**Affiliations:** ^1^Department of Prosthodontics, College of Dentistry and Dental Clinics, The University of Iowa, Iowa City, Iowa, USA; ^2^Department of Prosthodontics, School of Dentistry, Universidad de Especialidades Espiritu Santo, Samborondón, Guayas, Ecuador

## Abstract

Complete dentures often present significant challenges regarding precision and stability. With advances in 3D printing technologies and combined analog–digital workflows, new approaches have been developed that optimize both clinical efficiency and functional and aesthetic outcomes. This case report presents the treatment of a 70-year-old completely edentulous patient that was managed using an analog–digital workflow, with the prosthesis fabricated through 3D printing. Preliminary impressions were taken using alginate for the lower jaw and digital scanning for the upper jaw. Base plates were designed and 3D-printed, over which wax rims were placed for the determination of vertical dimension and centric relation. Subsequently, border molding was performed with the base, and a functional impression was taken using light body silicone. After obtaining the final impressions, a prototype printed in biocompatible resin was tested to evaluate occlusal and esthetic adjustments. Finally, the definitive prosthesis was 3D-printed using a high-precision printer. The process significantly reduced both clinical and laboratory time, with increased precision in denture adaptation and occlusion. The patient expressed excellent satisfaction with the final result, reporting improved comfort, functionality, and aesthetics compared to previous experiences with conventional dentures. This treatment approach, by integrating analog techniques with digital technologies, optimized both treatment efficiency and predictability.

## 1. Introduction

Conventional complete dentures continue to be the most widely used treatment for edentulous patients, primarily due to their cost-effectiveness and accessibility compared to implant-supported prostheses [1]. The fabrication of conventional complete dentures follows well-established clinical and laboratory protocols designed to optimize retention, stability, and function [2]. Traditional fabrication techniques, although time-consuming, provide several advantages, including the ability to capture detailed anatomical landmarks, accommodate functional movements through border molding, and achieve an individualized occlusal scheme tailored to the patient's needs. For this technique, materials such as alginate and polyvinyl siloxane may cause discomfort for patients, particularly those with a sensitive gag reflex [3].

Despite the long-standing success of conventional complete dentures, advancements in prosthodontics have introduced alternative workflows that aim to enhance clinical efficiency and patient experience [4]. The integration of digital technologies has provided new possibilities for impression-taking, denture design, and fabrication, addressing some of the limitations associated with traditional techniques [5]. While conventional methods continue to be widely utilized, particularly in complex cases requiring precise occlusal and anatomical considerations, the growing emphasis on accuracy, reduced clinical visits, and improved patient comfort has driven interest in digital workflows [6]. Digital technology has transformed prosthodontics by streamlining clinical procedures, minimizing material-related discomfort, and enhancing the reproducibility of prosthetic outcomes [5].

The implementation of intraoral scanners (IOSs) has significantly advanced denture fabrication by providing a noninvasive and highly precise method for capturing the morphology of edentulous arches [7]. In contrast to conventional impressions that may apply pressure to soft tissues, IOS enables the acquisition of truly mucostatic impressions, preserving the natural anatomy of the residual ridge [8]. This technique is particularly beneficial for patients with flabby or resorbed ridges, as it minimizes the risk of tissue distortion and supports the preservation of residual ridge morphology [9]. Nevertheless, a common challenge associated with IOS is the difficulty in replicating functional border molding, a critical factor in ensuring denture retention and stability. To address this limitation, recent innovations have focused on integrating digital techniques that simulate functional border molding, thereby enhancing the adaptation and performance of digitally fabricated dentures [8].

The integration of three-dimensional (3D) printing into prosthesis fabrication stands as one of the most transformative innovations in digital dentistry. This additive manufacturing technology facilitates the rapid and precise production of custom trays, trial dentures, and definitive prostheses, ensuring exceptional accuracy and fit [10]. Its capacity to create multimaterial components, such as resin-based denture bases and artificial teeth, facilitates personalized treatment solutions tailored to individual patient needs [5]. Additionally, 3D printing reduces material waste and optimizes laboratory workflows, thereby decreasing production time and associated costs [11]. By enhancing efficiency without compromising quality, this technology plays a pivotal role in modern prosthodontics, complementing intraoral scanning and software design to optimize the fabrication of complete dentures with improved functional and aesthetic outcomes [2].

This article is aimed at presenting a hybrid workflow that integrates conventional and digital techniques for complete denture fabrication. By combining the detailed impression-taking of traditional methods with the precision and efficiency of digital technologies, this approach seeks to optimize denture fit, retention, and patient comfort while reducing clinical and laboratory time. The synergy between analog impression techniques and digital design enables a more predictable and streamlined process, ensuring that the final prosthesis meets both functional and esthetic requirements.

## 2. Case Presentation

### 2.1. Patient Background and Clinical Evaluation

A 70-year-old edentulous female patient presented to the clinic with complaints of poor denture retention and impaired masticatory function. She reported difficulty in chewing and discomfort while wearing her current dentures, which had been fabricated several years ago using conventional methods. The patient expressed concerns about the esthetics and stability of her current prosthesis and sought a more advanced, long-lasting solution.

Upon clinical examination, the patient displayed a well-formed maxillary residual ridge, which provided a favorable foundation for denture support. However, the mandibular ridge exhibited moderate resorption, presenting challenges in achieving optimal retention and stability. The patient had no significant medical history or contraindications for dental treatment. Given her dissatisfaction with her previous dentures and the anatomical limitations observed, a hybrid analog–digital workflow was selected to enhance the precision and functionality of her new prosthesis.

### 2.2. Treatment Plan and Execution

**Figure 1:**
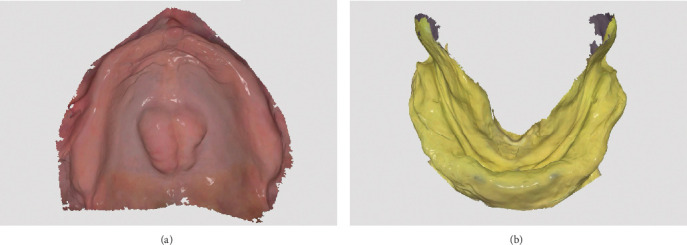
Digital and analog impression techniques. (a) Intraoral scan of the edentulous maxillary arch. (b) Digital scan of the mandibular impression.

To initiate the treatment, an intraoral scan of the maxillary arch was performed using a high-precision IOS (Trios 3; 3Shape, Copenhagen, Denmark) (Figure 1a). This allowed for the detailed capture of the maxillary anatomy without exerting pressure on the soft tissues. For the mandibular arch, an analog impression was taken using a frame cut back (FCB) tray, which had been 3D-printed using biocompatible material. The tray was filled with irreversible hydrocolloid (Cavex cream; Cavex, Haarlem, Netherlands), ensuring accurate anatomical reproduction. The mandibular impression was subsequently scanned using the same IOS (Figure 1b), enabling the digital conversion of both upper and lower arch data.

### 2.3. Digital Design and Custom Tray Fabrication

**Figure 2:**
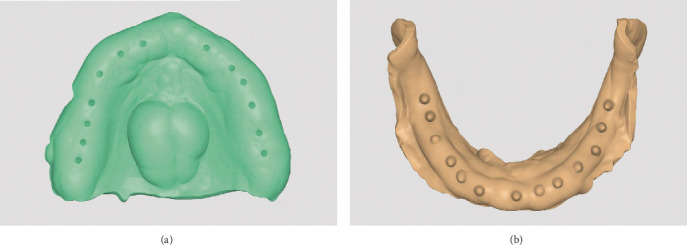
Custom tray design for maxillary and mandibular arches. (a) Digitally designed custom tray for the maxillary arch. (b) Digitally designed custom tray for the mandibular arch.

The acquired digital files were processed using specialized design software (Meditlink; Medit, Seoul, South Korea), where custom trays were designed for each arch. The maxillary custom tray was designed to incorporate essential anatomical landmarks, including the hamular notches and the posterior palatal seal, ensuring a proper extension for retention (Figure 2a). The mandibular tray design carefully accounted for the retromolar pad and buccal shelf, avoiding unnecessary impingement on the buccal frenum and mylohyoid ridge (Figure 2b). The trays were subsequently fabricated using a 3D printer (Model V2 Resin; Formlabs, Massachusetts, United States) and prepared for clinical use.

### 2.4. Wax Rim Adjustment and Occlusal Plane Verification

During the second appointment, wax rims were attached to the custom trays based on average occlusal values to determine the optimal vertical dimension of occlusion. The trays were placed intraorally to verify the occlusal plane orientation, lip support, and smile line. Adjustments were made as necessary to ensure a natural and balanced appearance. Key anatomical landmarks, such as the midline, canine lines, and incisal plane, were carefully evaluated to refine the positioning of the wax rims.

### 2.5. Border Molding and Final Impression

**Figure 3:**
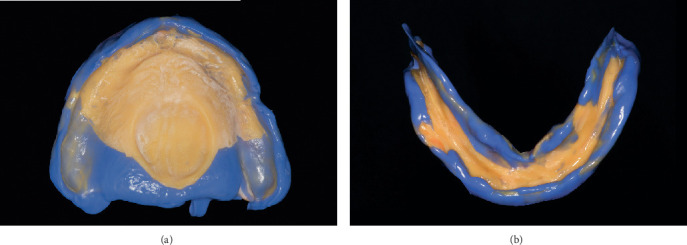
Functional border molding techniques. (a) Border molding of the maxillary tray. (b) Border molding of the mandibular tray.

Border molding was performed using a heavy-body vinyl polysiloxane (Heavy Body VPS; Plastcare, California, United States) to capture dynamic functional movements of the soft tissues. For the maxillary tray, the mucosa was gently retracted, and the patient was guided to perform controlled labial frenum movements during insertion (Figure 3a). In the mandibular arch, the patient was instructed to perform tongue movements, including side-to-side excursions and pressing the tongue against the posterior maxilla (Figure 3b). These functional movements facilitated the adaptation of the material to the dynamic oral environment, ensuring an optimal seal and improved prosthesis retention.

**Figure 4:**
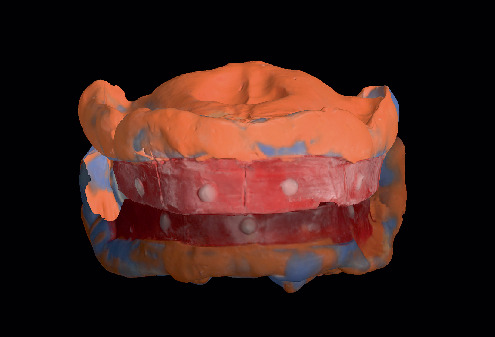
Final impression with vinyl polysiloxane (VPS) material and subsequent digital processing.

Following the border molding process, a final wash impression was made using light-body VPS (Light Body VPS; Plastcare USA, California, United States). This step allowed for the capture of fine details essential for the fabrication of a well-adapted denture base. The final impressions were digitized using the same IOS (Trios 3; 3Shape, Copenhagen, Denmark) and processed for CAD-based denture design (Figure 4).

### 2.6. Trial Denture Fabrication

**Figure 5:**
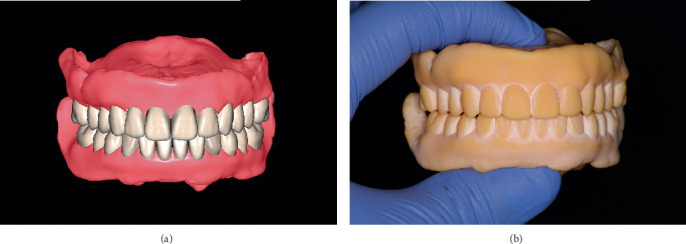
Digital design and 3D-printed trial denture. (a) Digital design of the complete denture. (b) 3D-printed monoblock trial denture.

The digital workflow proceeded with the design of the complete dentures using specialized CAD software (DentalCAD; Exocad GmbH, Darmstadt, Germany) (Figure 5a). Functional and esthetic elements were meticulously integrated to ensure an accurate replication of the captured intaglio surfaces and borders. The finalized design was exported for fabrication, and a monoblock trial denture was 3D-printed using a biocompatible resin (Soflex prototype; W2P Engineering, Vienna, Austria) (Figure 5b). During the third appointment, the trial denture was delivered, allowing the patient to evaluate its comfort, esthetics, and function over a 1-week period. Feedback was gathered, and necessary modifications were implemented to optimize fit and performance.

### 2.7. Final Denture Fabrication and Delivery

**Figure 6:**
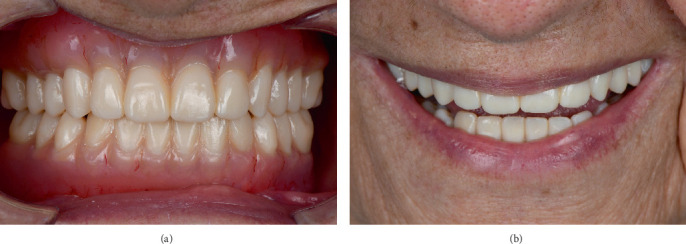
Final 3D-printed denture and follow-up outcome. (a) Definitive 3D-printed complete denture. (b) Posttreatment follow-up at 6 months.

Following approval of the trial denture, the definitive prosthesis was fabricated in two separate components: a pink base resin (Denture Base LP; Formlabs, Massachusetts, United States) and A1 shade resin teeth (Denture Teeth A1; Formlabs, Massachusetts, United States). These components were printed, assembled, and finalized for delivery at the fourth appointment (Figure 6a). Pressure-indicating paste was applied to identify and relieve any sore spots, ensuring patient comfort. The patient received detailed maintenance instructions, and follow-up visits were scheduled at 1 week, 1 month, and 6 months postdelivery. The clinical workflow was completed in four appointments: (1) initial impressions and digital scan, (2) wax rim try-in and occlusal registration, (3) trial denture evaluation, and (4) final denture delivery and adjustments.

During these follow-ups, adjustments were made as needed, and the patient reported significant improvements in mastication and esthetics, confirming the success of the treatment. The clinical team meticulously assembled the final denture components and delivered the prosthesis during the fourth appointment. Minor adjustments were carefully performed to enhance both comfort and function. The patient reported a significant enhancement in comfort and function, demonstrating the effectiveness of the hybrid analog–digital workflow in achieving a high-quality prosthetic outcome (Figure 6b).

## 3. Discussion

Conventional workflows for complete denture fabrication offer several well-established advantages, particularly in capturing detailed anatomical and functional information. The use of traditional impression materials, such as alginate and polyvinyl siloxane, allows for precise reproduction of soft tissue contours and functional movements essential for denture retention [2]. Additionally, border molding techniques ensure that the denture borders conform to the patient's vestibular anatomy, enhancing stability during mastication and speech [8]. The manual process also enables clinicians to make real-time adjustments, ensuring optimal fit and comfort. Despite advancements in digital technology, these analog techniques remain indispensable for achieving accurate impressions and predictable clinical outcomes [12].

Digital workflows significantly enhance the efficiency of complete denture fabrication by reducing both chair time and the number of required appointments [13]. IOSs eliminate the need for conventional impressions, minimizing the discomfort and time associated with impression taking [14]. Additionally, digital design software allows for faster customization and adjustment of denture components, streamlining the laboratory process [11]. The ability to 3D print trial dentures and final prostheses further accelerates production, enabling quicker turnaround times. Studies have shown that digital workflows can reduce the number of clinical visits from five to as few as two or three while also minimizing the need for postinsertion adjustments, enhancing both patient satisfaction and clinical productivity [6].

Integrating hybrid workflows that encompass both analog and digital techniques presents various challenges that clinicians must overcome to achieve optimal outcomes. The adoption of digital technology requires practitioners to develop advanced skills in intraoral scanning, design software, and 3D printing processes, representing a significant learning curve [15]. Furthermore, the initial investment in essential equipment, such as scanners, design software, and 3D printers, can be substantial, potentially limiting accessibility for certain dental practices [16]. Additionally, the selection of printable resins is critical, as material properties directly influence the mechanical strength, esthetics, and longevity of the final prosthesis, underscoring the importance of careful material selection to ensure optimal clinical outcomes [17].

The fusion of analog and digital techniques offers the best of both worlds, combining the precision of digital technology with the clinical adaptability of conventional methods [14]. Hybrid workflows allow clinicians to perform critical steps, such as border molding and impression-making, in analog form while utilizing digital technology for design and fabrication [8]. This approach maintains essential clinical protocols while enhancing accuracy and efficiency. Patients benefit from improved fit and function, while clinicians can achieve greater consistency in denture outcomes [18].

This technique not only reduces clinical visits and laboratory processing time but also minimizes patient discomfort during impression-taking [7]. The hybrid analog–digital workflow represents a promising approach, particularly in cases where digital scanning of the mandibular arch presents challenges in capturing anatomical details. By integrating traditional border molding and impression techniques with digital design and 3D printing, clinicians can achieve enhanced accuracy and retention, ensuring the denture conforms precisely to the patient's soft tissue anatomy. Additionally, the workflow improves consistency in denture fabrication while allowing for greater customization, supporting its adoption as an efficient and reliable alternative to fully digital or conventional methods.

## 4. Conclusion

The hybrid workflow integrates conventional mucostatic impressions with digital design and fabrication, enhancing denture retention, stability, and precision. This approach reduces clinical visits, minimizes patient discomfort, and ensures consistent prosthetic outcomes, offering an efficient and reliable solution for edentulous patients with anatomical scanning challenges, optimizing both functional and esthetic results.

## Data Availability

The data that support the findings of this case report are available from the corresponding author upon reasonable request.
